# Rho GTPase-dependent plasticity of dendritic spines in the adult brain

**DOI:** 10.3389/fncel.2013.00062

**Published:** 2013-05-23

**Authors:** Assunta Martino, Michele Ettorre, Marco Musilli, Erika Lorenzetto, Mario Buffelli, Giovanni Diana

**Affiliations:** ^1^Department of Therapeutic Research and Medicines Evaluation, Istituto Superiore di SanitàRoma, Italy; ^2^Department of Neurological, Neuropsychological, Morphological and Motor Sciences, Section of Physiology, University of VeronaVerona, Italy; ^3^Center for Biomedical Computing, University of VeronaVerona, Italy; ^4^National Institute of NeuroscienceVerona, Italy

**Keywords:** dendritic spines, two-photon microscopy, Golgi staining, Rho GTPases, cytotoxic necrotizing factor 1, brain plasticity, mice

## Abstract

Brain activity is associated with structural changes in the neural connections. However, *in vivo* imaging of the outer cortical layers has shown that dendritic spines, on which most excitatory synapses insist, are predominantly stable in adulthood. Changes in dendritic spines are governed by small GTPases of the Rho family through modulation of the actin cytoskeleton. Yet, while there are abundant data about this functional effect of Rho GTPases *in vitro*, there is limited evidence that Rho GTPase signaling in the brain is associated with changes in neuronal morphology. In the present work, both chronic *in vivo* two-photon imaging and Golgi staining reveal that the activation of Rho GTPases in the adult mouse brain is associated with little change of dendritic spines in the apical dendrites of primary visual cortex pyramidal neurons. On the contrary, considerable increase in spine density is observed (i) in the basal dendrites of the same neurons (ii) in both basal and apical dendrites of the hippocampal CA1 pyramidal cells. While confirming that Rho GTPase-dependent increase in spine density can be substantial, the study indicates region and dendrite selectivity with relative stability of superficial cortical circuits.

## INTRODUCTION

Changes in the structure of synaptic connections underlie various neurological processes ranging from the development of neuronal circuitry to injury-related recovery and cognition (Hubel et al., 1977; Rakic et al., 1994; Florence et al., 1998; Jones and Pons, 1998; Lichtman and Colman, 2000; Keck et al., 2008). In particular, the rearrangement of neural networks produced by changes in dendritic spines has been associated with learning and memory processes (McGaugh, 2000; Chklovskii et al., 2004). Recent technological advances, including two-photon microscopy and transgenic mice overexpressing fluorescent proteins have made possible to image individual dendritic arbors and spines over long periods of time in living animals. By this technique, active generation of spines/protrusions was observed in the developing brain, when the wiring of the neural networks is being established (Lendvai et al., 2000; Grutzendler et al., 2002; Majewska et al., 2006; Holtmaat and Svoboda, 2009). Although several studies indicate that synaptic connectivity in adulthood can be continuously modified by learning-related tasks and environmental factors (Fox, 2002; Grossman et al., 2002; Knott et al., 2002; Yang et al., 2009), cortical circuits appear to be rather stable in the adulthood (Grutzendler et al., 2002; Majewska et al., 2006).

Spine structure changes through the reorganization of the actin cytoskeleton (Matsuzaki et al., 2004; Okamoto et al., 2004; Honkura et al., 2008). This latter is regulated by GTPases belonging to the Rho family (Van Aelst and Cline, 2004; Auer et al., 2011; Chen et al., 2012; Suo et al., 2012), a class of hydrolases ubiquitously expressed in eukaryotic cells that includes Rho, Rac, and Cdc42 subfamilies (Etienne-Manneville and Hall, 2002). Studies using neuronal cell lines revealed that both Rac1 and Cdc42 are required for neurite formation and outgrowth; conversely, Rho activation suppresses neurite outgrowth and induces neurite retraction (Luo, 2000; Lorenzetto et al., 2013). Yet, most of the aforementioned studies were carried out in cultured neural cells, and there is little evidence that Rho GTPase signaling in the brain be associated with changes in neuronal morphology (Cerri et al., 2011; Suo et al., 2012). In addition, not much is known about the regional and subcellular distribution of Rho GTPase-dependent plasticity*.*

Cytotoxic necrotizing factor 1 (CNF1), a 114 kDa protein toxin produced by *Escherichia coli*, produces a rearrangement of the cytoskeleton in intact cells through permanent activation of Rho, Rac1, and Cdc42 (Boquet, 2001). This capability, previously observed in epithelial cells, extends to neurons (Diana et al., 2007; Malchiodi-Albedi et al., 2012). In the present work, we sought to determine whether the activation of cerebral Rho GTPases through CNF1 is followed by changes in neuronal morphology in the adult mouse brain. We analyzed the fine morphology of hippocampal CA1 and V1 visual cortex pyramidal neurons by Golgi-Cox staining. Changes in the structure of neuronal processes in V1 area of the visual cortex were also followed by chronic *in vivo* two-photon fluorescence imaging. The functional relevance of the changes observed was investigated by *in vitro* recording of activity-dependent plasticity phenomena, such as paired-pulse facilitation (PPF) and long-term potentiation (LTP). Altogether, our results indicate that Rho-dependent structural plasticity is substantial and widespread in hippocampal CA1 pyramidal neurons and weak in apical dendrites of V1 pyramidal neurons, thus suggesting a regional and dendritic selectivity.

## MATERIALS AND METHODS

### MORPHOMETRICAL ANALYSIS OF Golgi-Cox STAINED SECTIONS

#### Animals

The experiments were carried out on eight male C57BL/6J mice (Harlan Italy, S. Pietro al Natisone, Udine, Italy) aged 3 months at the time of the treatment. The mice were housed at 21 ± 1°C at constant humidity (55%) and in a 12/12 h dark–light cycle, with light phase from 08:00 to 20:00. Food and water were provided *ad libitum*. The use and care of the animals was compliant with the Italian law (DL 116/92) and with the guidelines of the European Communities Council (1986).

#### Intracerebroventricular injections

Under general anesthesia (sodium pentobarbital, 50 mg kg^-^^1^ i.p.), a 27G needle mounted on a 25 μl Hamilton microsyringe was placed in the right lateral cerebral ventricle with a stereotaxic technique (coordinates from bregma and skull bone: A/P = -1.0 mm, L/M = +1.0 mm, D/V = -3.0 mm; Paxinos and Franklin, 2004). The mice were injected 3.3 μl of 1.0 fmol kg^-^^1^ CNF1 (GenBank X70670.1, *n* = 4) or vehicle (20 mM TRIS-HCl buffer, pH 7.5; *n* = 4). Five minutes post-injection, the needle was removed and the surgical wound sutured. From this time on, the mice were housed in individual cages and monitored for general conditions for the following 7 days.

#### Golgi-Cox impregnation of brain tissue

Ten days post-injection, the animals were deeply anesthetized with sodium pentobarbital (50 mg kg^-^^1^ i.p.) and perfused transcardially with 150 ml of saline at room temperature. Brains were prepared for Golgi-Cox staining as previously described (Glaser and Van der Loos, 1981). Briefly, the brains were removed from the skull and immediately stored in Golgi-Cox solution (1% potassium dichromate/1% mercuric chloride/0.8% potassium chromate) at room temperature for 6 days. Subsequently, they were transferred into a sucrose solution (30%) and stored at room temperature for 3 days. Then, coronal sections of the brains (100 μm thick) were prepared using a vibroslicer. The sections were mounted on gelatinized slides and stained according to the procedure previously described (Gibb and Kolb, 1998). The slides were covered and left drying at room temperature overnight. On the following day, the brain sections were observed under light microscope (Zeiss Axioskop, Germany) at 4×–100× magnification, the latter in oil immersion.

#### Morphological analysis

**FIGURE 1:**
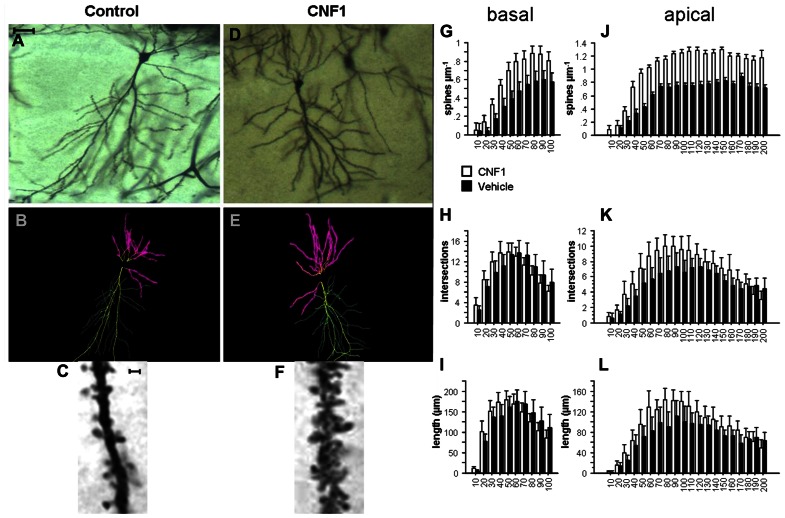
**Rho GTPase activation increases spine density and dendrite branching in hippocampal CA1 pyramidal neurons.** **(A,D)** Photomicrographs (scale bar 100 μm); **(B,E)** Neurolucida tracings; **(C,F)** detail of apical dendrite (scale bar 2 μm) of representative Golgi stained neurons in hippocampal CA1 of C57BL/6J mice treated 10 days before histology either with vehicle (control, **A,C**) or 1.0 fmol kg^-^^1^ CNF1 i.c.v. **(D,F)**. Spine density **(G,J)**, number of intersections **(H,K)**, and average dendrite length **(I,L)** in basal **(G,I)** and apical **(J,L)** dendrites are plotted by distance from cell soma (μm). Mean ± SEM; *n* = 16 in each group.

**FIGURE 2:**
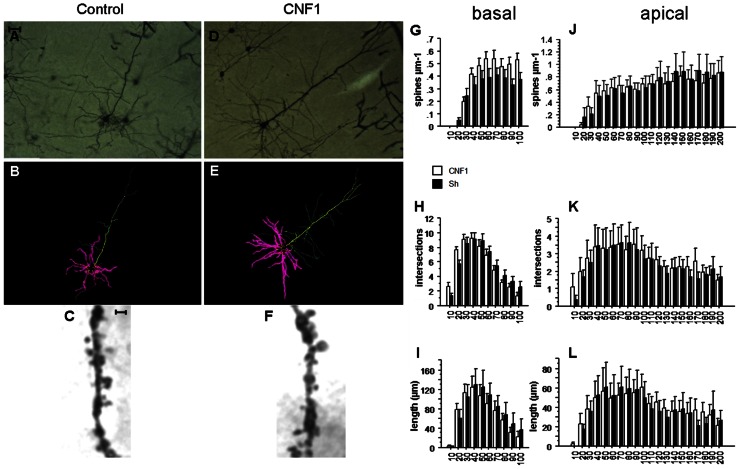
**Rho GTPase activation increases spine density in basal but not apical dendrites ofV1 visual cortex.** **(A,D)** Photomicrographs (scale bar 100 μm); **(B,E)** Neurolucida tracings; **(C,F)** detail of apical dendrite (scale bar 2 μm) of representative Golgi stained neurons in V1 visual cortex of C57BL/6J mice treated 10 days before histology either with vehicle (control, **A,C**) or 1.0 fmol kg^-^^1^ CNF1 i.c.v. **(D,F)**. Spine density **(G,J)**, number of intersections **(H,K)**, and average dendrite length **(I,L)** in basal **(G,I)** and apical **(J,L)** dendrites are plotted by distance from cell soma (μm). Mean ± SEM; *n* = 16 in each group.

Measurements were performed in fully impregnated pyramidal neurons displaying dendritic tree without obvious truncations. Sixteen neurons from hippocampal CA1 (A/P stereotaxic coordinates from bregma: -1.5 to -2.5 mm; two neurons/hemisphere/mouse/treatment; **Figures 1A–F**) and 16 from V1 visual cortex with cell soma in the layer V (A/P stereotaxic coordinates from bregma: -2.6 to -3.2 mm; two neurons/hemisphere/mouse/treatment; **Figures 2A–F**; Paxinos and Franklin, 2004) were analyzed.

The neurons were traced and analyzed using Neurolucida software (MicroBrigthtField, Williston, VT, USA). The tracings (see **Figures 1B,E** and **2B,E**) were carried out on images obtained from a camera (Optronics, Chelmsford, MA, USA) connected to the microscope. The morphological analysis (**Figures 1G–L** and **Figures 2G–L**) was performed by two different operators that were blind to the treatment of the animal.

***Dendrites length and branching*** Dendrites were assigned to three different classes: apical, basal, and oblique. For the branching analysis, the basal dendrites were classified using a “zcentrifugal” method (Uylings et al., 1986). The branches arising from the soma were classified as order I, the bifurcations which arose from the branches of the order I were named as order II, and so on. The dendrites that belonged to the apical ones were defined as oblique dendrites. The analyzed dendrites did not have truncated ramifications and were checked for continuity with the neuron to avoid misattribution to neighboring neurons and erroneous dendritic spine counting. Neurons were selected so that they had an order of ramification for the basal dendrites ≥III and a number of branches detaching from the soma of at least of three dendrites. The neurons were traced along their entire length. Sholl analysis was performed to determine the cumulative dendritic length and the number of dendritic intersections within concentric spheres centered on the neuron soma.

***Spine density*** Neurons were first identified under low-magnification (20×). Subsequently, they were traced under high-magnification (100×). All protrusions showing a clear connection to the dendritic shaft were considered as spines and traced for subsequent analysis. The spine density was defined as the number of spines on 1 μm of dendritic length. Spines were counted on the whole and entire apical and oblique dendritic lengths but only on basal dendrites with order III of branching or higher. Sholl analysis was performed to determine spine densities by distance from the neuron soma.

### IN VIVO SPINE IMAGING

#### Animals and surgical preparation

Male 3/4-month old C57BL/6J transgenic mice, in which a Thy-1 promoter drives the expression of green fluorescent protein (GFP, line M) in a subset of cortical neurons (Feng et al., 2000) were used for the study. Surgical procedures were performed as described previously (Holtmaat et al., 2009; Laperchia et al., 2013). Briefly, mice were deeply anesthetized with an i.p. injection of avertin (400 mg kg^-^^1^). Before surgery, dexamethasone (2 mg kg^-^^1^ i.m.) and carprofen (0.3 ml of a 0.50 mg ml^-^^1^ solution i.p.) were injected to prevent cerebral edema and inflammation and to limit pain.

Primary visual cortex was identified on the basis of stereotaxic coordinates (Paxinos and Franklin, 2004). The skull overlying visual cortex was removed, taking care not to damage the dura. The dura was covered with a coverglass (5 mm diameter, 0.15 mm thickness, Warner Instruments, CT, USA), sealed in place with dental acrylic glue.

Imaging was started 15–20 days after surgery. Between imaging sessions, animals were housed individually in plastic cages. Fifteen–twenty days after cranial window implantation, 15 mice were assigned to four groups, 3 of which received i.c.v. injection of CNF1 (*n* = 4), CNF1 C866S (a recombinant molecule in which the enzymatic activity was abolished by replacing serine to cysteine at position 866; *n* = 4; Schmidt et al., 1998) or vehicle (20 mM TRIS-HCl buffer, pH 7.5; *n* = 4). The injections were performed as described above (“Intracerebroventricular injections”). A fourth group of untreated mice (*n* = 3) was used as additional control.

#### Imaging

Animals were anesthetized with isoflurane. *In vivo* images of GFP-expressing neurons were acquired by a two-photon laser scanning microscope (2PLSM; TCS-SP5, Leica Microsystems, Germany) equipped with a Ti:Sapphire tunable laser, (680–1080 nm; Chameleon Ultra, Coherent Inc, CA, USA). The objective was water immersion HCX APO L 20x/1.00 NA (Leica Microsystems, Germany). The GFP was excited at 920 nm and the signal was collected by non-descanned detectors in the range 500–530 nm. In each animal, the apical dendritic tufts of layer V pyramidal neurons were imaged for 16 days. In each session, the dendritic tufts were localized using as a reference the vascular pattern of the cortical region, and then using low-magnification 2PLSM imaging to identify the cell of interest by the unique branching pattern of its apical dendrites. For high-magnification spine imaging, 7–10 fields were selected for each cell. Image stacks consisted of sections (512 × 512 pixels; 90 nm/pixel, pixel dwell time 2.5 μs) collected in 1 μm z-step size. Care was taken to achieve almost identical fluorescence levels across imaged regions and imaging sessions. All images in the figures are maximum intensity projections (MIPs) of z-stacks.

#### Spine turnover analysis

A total of 2830 spines were tracked in time-lapse images on day 1 before treatment administration and subsequently on day 5, 10, and 15 post-injection. All clear protrusions emanating laterally from the dendritic shaft were measured. Evaluation of spine appearance/disappearance was based on the following criteria: spines were considered as lost if they disappeared into the haze of the dendrite, whereas spines were considered as gained if they showed clear protrusion from the dendrite.

Turnover ratios (TORs), i.e., the fraction of spines appearing and disappearing from an imaging session to the following one were calculated as (number gained + number lost)/(2 × total number). The time-dependent survival function was calculated as SF(*t*) = *N*(*t*)/No, where No is the number of spines at *t* = 0, and *N*(*t*) is the number of spines of the original set surviving after time *t*. By definition, SF(*t*) is a monotonically decreasing function of time, and SF(0) = 1 (Holtmaat et al., 2005).

### HIPPOCAMPAL AND CORTICAL SLICE PREPARATION AND ELECTROPHYSIOLOGY

Two groups of C57BL/6J mice, treated with either 1.0 fmol kg^-^^1^ CNF1 or vehicle were used for *in vitro* electrophysiology experiments. Intracerebroventricular administration of the test solution was carried out as described above. Ten–eighteen days post-treatment, the mice were deeply anesthetized with urethane (1.5 g kg^-^^1^ i.p.) and decapitated. The brains were removed and the hippocampus and primary visual cortex were isolated. Transverse hippocampal or cortical slices, 400 μm thick, were cut with a tissue chopper (The Mickle Laboratory Engineering Co. Ltd., Gomshall, Surrey, UK), transferred to an incubation glass chamber containing artificial cerebrospinal fluid (ACSF) saturated with a gas mixture of 95% O_2_ and 5% CO_2_ and maintained at room temperature for at least 2 h. ACSF is a water solution (pH 7.4) containing (mM): 126 NaCl, 3.5 KCl, 1.2 NaH_2_PO_4_, 25 NaHCO_3_, 2 CaCl_2_, 1.3 MgCl_2_, 11 glucose. For electrophysiological experiments, slices were transferred to a submerged-type recording chamber, placed about 100 μm below the surface and perfused with oxygenated ACSF (24 ± 1°C) with a peristaltic pump (Gilson Minipuls3, WI, USA) at a constant flow rate (2.5–3 ml min^-^^1^). An electrode (stainless steel, 250 μm diameter, tapered tip size 8°, 5 MΩ; A-M Systems Inc., WA, USA) was placed into the *stratum radiatum* within the CA1 area to stimulate the Schaffer’s collateral-commissural fibers or in the layer IV of the V1 area of the visual cortex. Glass micropipettes (OD 1.0 mm, ID 0.7 mm, 1.5–2 MΩ) filled with ACSF were placed in the hippocampal dendritic layer of the CA1 area or in the layer III of V1 for extracellular recording of field excitatory post-synaptic potentials (fEPSPs). The depth of the electrodes was adjusted in order to maximize the height of the fEPSPs, which were evoked by regular stimulation (0.033 Hz; squared waves, 100 μs; constant current). The responses were amplified 1000 times and filtered at 10 kHz (L-C low pass filter, 40 dB/decade). The signals were then sampled at 40 kHz, digitized and stored on disk for subsequent off-line analysis.

Ten minutes before the induction of LTP and 1 h after LTP, neurotransmission was studied by recording input–output curves, i.e., the responses produced by 11 consecutive stimuli of increasing intensity (0–200 μA in steps of 20 μA). Stimulus intensity used throughout LTP experiments was selected so that fEPSP initial slopes ranged from 40 to 60% of the maximum obtained in the first input–output curve. For analysis, only slices that reached a steady response in 30 min were used. LTP was induced by three consecutive theta-burst stimulations (TBS, inter-stimulation interval = 30 s; 10 trains of 4 stimuli at 100 Hz, baseline intensity; inter-train interval = 200 ms) and recorded for at least 1 h. In non-potentiated slices, PPF was elicited at six interpulse intervals (25, 50, 100, 200, 300, and 400 ms, stimulation intensity selected as for LTP experiments). Data were entered into analysis as a single subject, and therefore reflect individual mice.

### STATISTICAL ANALYSIS

Data were analyzed by analysis of variance (ANOVA). Repeated measurement designs were implemented for the analysis of (a) Golgi stained sections morphometrical data, using the distance from soma as “within subjects” factor; (b) time-lapse two-photon microscopy data, using time of imaging as “within subjects” factor; (c) fEPSP input/output curves. LTP and PPF experiments were analyzed by analysis of covariance (ANCOVA) for repeated measurements, using the average slope of baseline responses as covariate. All calculations were performed using Statistica^TM^ 5.0 or SPSS for Windows.

## RESULTS

### Rho GTPase ACTIVATION INCREASES SPINE DENSITY IN HIPPOCAMPAL CA1

Activation of Rho GTPases produces a substantial increase in spine density on both basal and apical dendrites of hippocampal CA1 pyramidal neurons. In particular, spine density on basal dendrites was 0.594 ± 0.027 and 0.376 ± 0.021 in CNF1-treated and vehicle-treated mice, respectively (spine/μm; mean ± SEM; *F*_1,29_ = 36.307, *P* < 0.0001). The different distribution of spine density by radius was statistically significant, as well (*F*_9,261_ = 5.494, *P* < 0.0001; **Figure 1G**).

On apical dendrites, spine densities were 1.016 ± 0.025 and 0.626 ± 0.018 in CNF1-treated and in vehicle-treated group, respectively (*F*_1,28_ = 112.004, *P* < 0.0001; **Figures 1C,F**). The distribution of spines by radius was also significantly different in the two groups (*F*_19,532_ = 4.419, *P* < 0.0001; **Figure 1J**). Spines increased considerably on segments that usually show little or no spine density, like the principal dendrite shaft, oblique dendrites, and the terminal segment of hippocampal apical dendrite.

### Rho GTPase ACTIVATION INCREASES DENDRITE BRANCHING IN HIPPOCAMPAL CA1

Intersections of CA1 pyramidal neurons apical dendrites were increased by CNF1 treatment. In treated group the average number of intersections was 6.597 ± 0.220, whereas in control group it was 5.133 ± 0.168 (*F*_1,28_ = 8.036, *P* = 0.0084). The difference was more pronounced in proximal dendrites (*F*_19,532_ = 2.791, *P* < 0.0001; **Figure 1K**).

In the same neurons, there was no difference in the average number of intersections of basal dendrites (9.957 ± 0.362 vs. 9.931 ± 0.410 in CNF1-treated and control group, respectively; *F*_1,28_ = 0.001, *P* = 0.9762). The trend in the number of intersections according to the distance from the cell soma (radius) is illustrated in **Figure 1H**. It appears that intersections are increased in the proximal dendrites, whereas they tend to decrease in the distal ones (*F*_9,252_ = 2.569, *P* = 0.0076; **Figure 1H**).

The trends in dendrite length by distance from cell soma match those obtained for the number of intersections. The average length of basal dendrites was not affected by treatment (125.222 ± 5.031 and 125.309 ± 5.4461 μm in CNF1 and control group, respectively; *F*_1,29_ = 0.0001, *P* = 0.9931). As for the number of intersections, CNF1 increased the length of proximal and reduced that of distal dendrites (*F*_9,261_ = 2.654, *P* = 0.0058; **Figure 1I**).

For apical dendrites, the average length was 90.581 ± 3.137 and 71.490 ± 2.611 in CNF1 and control group, respectively (*F*_1,28_ = 6.186, *P* = 0.0191). The average length of proximal but not distal sections of dendrites was increased by CNF1 treatment (*F*_19,532_ = 2.286, *P* = 0.0016; **Figure 1L**).

Altogether, the results show that the activation of Rho GTPases stimulates branching of the dendritic tree in the proximal sections of both apical and basal dendrites in hippocampal CA1. This finding suggests that Rho-dependent neuronal morphogenesis in the adult brain is not restricted to spines but it involves the dendritic tree.

### Rho GTPase ACTIVATION INCREASES SPINE DENSITY IN BASAL BUT NOT APICAL DENDRITES IN V1 VISUAL CORTEX

In basal dendrites of V1 visual cortex pyramidal neurons, the Rho GTPase-dependent increase in spine density was significant, though less sizeable than in hippocampal CA1. Spine densities were 0.368 ± 0.021 and 0.288 ± 0.021 in CNF1- and in vehicle-treated group, respectively (spine/μm; mean ± SEM; *F*_1,33_ = 1.735, *P* = 0.1968; difference by distance from cell soma: *F*_9,297_ = 3.316, *P* = 0.0007; effect of distance from cell soma: *F*_9,297_ = 65.250, *P* < 0.0001; **Figure 2G**). In particular, at 80–90 and 90–100 μm from the cell soma, spine density was significantly higher in CNF1-treated mice (*P* < 0.05).

On the contrary, differences in spine densities were not significant in apical dendrites of the same neurons. In CNF1-treated group the average spine density was 0.476 ± 0.015 and in the control group it was 0.517 ± 0.018 (spine/μm; mean ± SEM; *F*_1,34_ = 0.419, *P* = 0.5218 by repeated measurement ANOVA; **Figures 2C,F**; difference in spine density by distance from cell soma: *F*_19,646_ = 1.122, *P* = 0.3236; **Figure 2J**). Spines were unevenly distributed along the length of the dendrites (effect of distance from cell soma: *F*_19,646_ = 37.257, *P* < 0.0001; **Figure 2J**).

### Rho GTPase ACTIVATION DOES NOT AFFECT DENDRITE BRANCHING IN V1 VISUAL CORTEX

Intersections in apical dendrites were****2.575 ± 0.087 and 2.400 ± 0.092 in CNF1-treated and control group, respectively (*F*_1,34_ = 0.384, *P* = 0.5398; **Figure 2K**). No differences in total intersections were observed in the basal dendrites of CNF1-treated mice (5.570 ± 0.358 vs. 5.650 ± 0.311 in CNF1-treated and control group, *F*_1,28_ = 0.013, *P* = 0.9111; differences in intersections by distance from cell soma: *F*_9,252_ = 2.342, *P* = 0.0149; effect of distance from cell soma: *F*_9,252_ = 71.122, *P* < 0.0001; **Figure 2H**).

The average length of basal dendrites by distance from cell soma was 70.474 ± 3.921 in CNF1-treated and 76.472 ± 4.801 in control group (μm, *F*_1,27_ = 0.370, *P* = 0.5484; interaction dendrite length × radius: *F*_9,243_ = 1.552, *P* = 0.1306; **Figure 2I**). For apical dendrites, the average length was 40.054 ± 1.488 and 38.362 ± 1.796 in CNF1-treated and control group, respectively (*F*_1,34_ = 0.138, *P* = 0.7130; differences in the effects of treatment by radius: *F*_19,646_ = 0.701, *P* = 0.8196; **Figure 2L**).

### Rho GTPase ACTIVATION DOES NOT AFFECT SPINE DYNAMICS IN THE SUPERFICIAL V1 VISUAL CORTEX

**FIGURE 3:**
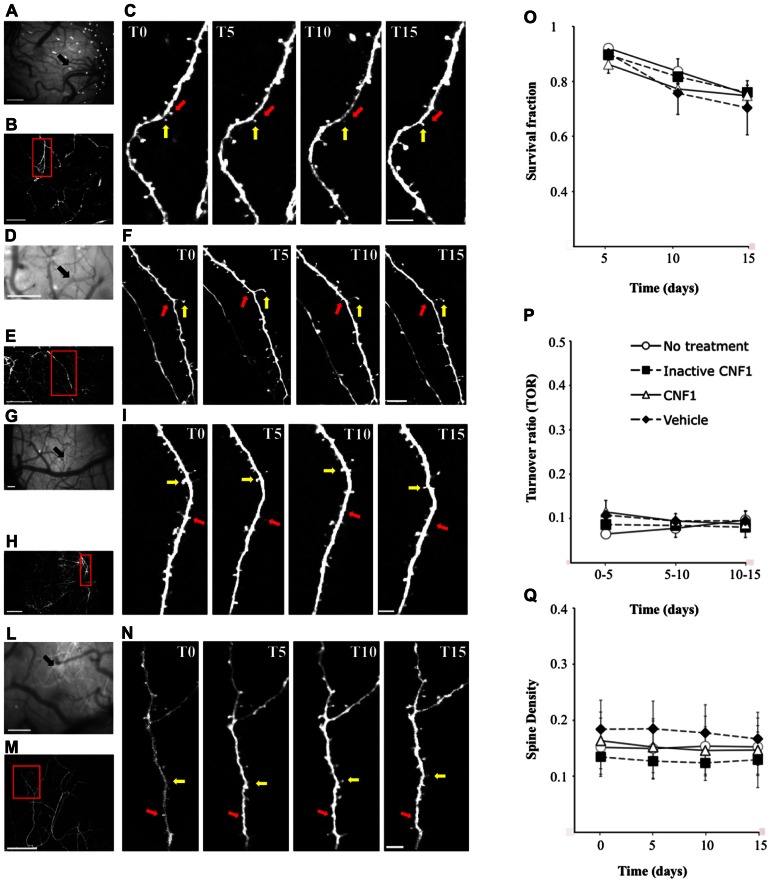
**Rho GTPase activation does not increase spine density and turnover in apical dendrites of primary visual cortex.** GFP-m mice were injected with CNF1 (1.0 fmol kg^-^^1^ i.c.v., *n* = 4), CNF1 C866S (1.0 fmol kg^-^^1^ i.c.v., *n* = 4), vehicle (20 mM TRIS-HCl buffer, pH 7.5, *n* = 4), or did not received any treatment (*n* = 3) and transcranial two-photon imaging in time-lapse of dendritic spines was performed. Examples of images collected from mice treated with CNF1 **(A,C)**, CNF1 C866S **(D,F)**, vehicle **(G,I)**, and without treatment **(L,N)**. **(A,D,G,L)** bright field views of the vasculature below cranial window. The arrows indicate the region where two-photon images were acquired. **(B,E,H,M)** low-magnification images of a layer V dendritic arbor in visual cortex. **(C,F,I,N)** time-lapse images of dendritic branches acquired 1 day before treatment (T0) and 5 (T5), 10 (T10), and 15 (T15) days after injection (from boxed regions in **B,E,H,M**, respectively). Figures show an example of persistent (yellow arrows) and transient spines (red arrows). Mean survival fraction **(O)**, mean turnover ratios **(P)** and mean spine density **(Q)** are plotted as a function of time. Scale bars = 200 μm **(A,D,G,L)**; 100 μm **(B,E,H,M)**; 5 μm **(C,F,I,N)**.

Although CNF1 does not affect the density of spines and branching of apical dendrites of layer V pyramidal neurons in V1 visual cortex, changes in the turnover of spines or dendrite morphology may have occurred that are not reflected in the morphometry of Golgi stained cells. For this reason, the stability of apical dendritic tufts under the effects of CNF1 was studied by repeated, time-lapse *in vivo* two-photon imaging (**Figures 3A, C**). A group of control mice was treated with CNF1 C866S, a recombinant CNF1 in which the enzymatic activity was abolished by substitution of serine with cysteine at position 866 (Schmidt et al., 1998; **Figures 3D,F**). A group of mice that received only vehicle injection and a group of untreated, age-matched mice were used as additional controls (**Figures 3G,I** and **L,N**, respectively).

We monitored the overall effects of Rho GTPase activation during the first two weeks after treatment. We did not see any change in the morphology of the superficial processes of layer V pyramidal neurons of V1 visual cortex. We then evaluated dendritic spine turnover, stability, and density.

Individual spines observed in the first imaging session, a total 860, 773, 847, and 350 spines in CNF1-, CNF1 C866S-treated, vehicle injected, and untreated group, respectively, were followed up. The fraction of spines surviving until the last imaging session (survival fraction, Sf) was calculated. No significant differences were observed among the four groups in Sf (CNF1: 79.0 ± 2.3%, *n* = 4; CNF1 C866S: 82.4 ± 2.1%, *n* = 4; vehicle: 78.4 ± 6.0%, *n* = 4; untreated mice: 83.0 ± 3.0%, *n* = 3; mean ± SEM; *F*_6__,__22_ = 0.654, *P* = 0.686; **Figure 3O**).

A subpopulation of spines appeared and disappeared across imaging sessions (**Figures 3C,F,I,N**). This phenomenon was observed in all groups. The rate of spine turnover (TOR) was slightly higher in CNF1 treated mice than in the controls, even though not significantly (CNF1: 9.8 ± 2.5%, *n* = 4; CNF1 C866S: 8.3 ± 1.6%, *n* = 4; vehicle: 9.2 ± 1.8%, *n* = 4; untreated mice: 8.0 ± 1.5%, *n* = 3; mean ± SEM; *F*_6__,__22_ = 1.764, *P* = 0.153; **Figure 3P**).

Spine density (average of all time points) was comparable among the four treatment groups (CNF1: 0.154 ± 0.045, *n* = 4; CNF1 C866S: 0.130 ± 0.035, *n* = 4; vehicle: 0.170 ± 0.050%, *n* = 4; untreated mice: 0.152 ± 0.050, *n* = 3; spines/μm; mean ± SEM; *F*_9__,__33_ = 1.291, *P* = 0.279; **Figure 3Q**).

In conclusion, the activation of Rho GTPases did not induce changes in the superficial dendritic tree of V1 pyramidal neurons.

### Rho GTPase ACTIVATION ENHANCES GLUTAMATERGIC NEUROTRANSMISSION AND LONG-TERM POTENTIATION IN HIPPOCAMPAL CA1 BUT NOT IN V1 VISUAL CORTEX

Input–output curves were analyzed by a one-way ANOVA for repeated measurements, in which treatment (“control,” “CNF1”) was “between-subjects” factor and both the effects of LTP “pre-LTP” and “post-LTP”) and the trend of responses at increasing stimulation intensity (11 levels, “0” to “200”) were "within-subjects” factor.

**FIGURE 4:**
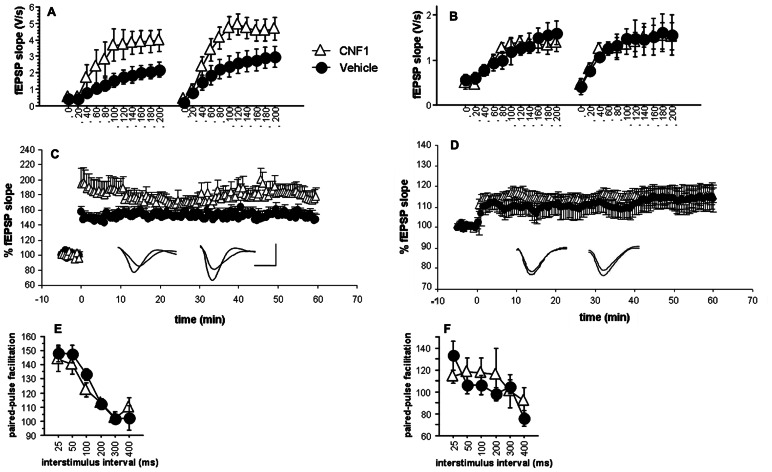
**Enhancement of glutamatergic neurotransmission and long-term potentiation in hippocampal CA1 but not inV1 visual cortex after Rho GTPase activation.** Input–output curves of fEPSPs recorded 10 min before and 1 h after induction of long-term potentiation obtained by three consecutive theta-burst stimulation (TBS, inter-stimulation interval = 30 s; 10 trains of 4 stimuli at 100 Hz, baseline intensity; inter-train interval = 200 ms) in CNF1-treated (1.0 fmol kg^-^^1^ i.c.v.), and control (vehicle-treated) C57BL/6J mice (control, *n* = 6; CNF1, *n* = 8); **(A)** hippocampal CA1 and **(B)** V1 visual cortex. Time course of fEPSP slopes pre- and post-TBS and representative traces (left, control; right, CNF1; horizontal scale bar: 1.5 ms; vertical scale bar: 1.5 mV) in **(C)** hippocampal CA1 (control, *n* = 12; CNF1, *n* = 13) and **(D)** V1 visual cortex (control, *n* = 12; CNF1, *n* = 10). Paired-pulse facilitation at different interstimulus intervals (control: *n* = 6, CNF1: *n* = 6) in **(E)** hippocampal CA1 and **(F)** V1 visual cortex. All recordings were performed 10–18 days post i.c.v. injections. Data are mean ± SEM.

The ANOVA on the initial slopes of the fEPSP in the hippocampus showed that the responses recorded during the generation of input–output curves were significantly different in control and in CNF1-treated mice (*F*_1,12_ = 5.003, *P* = 0.0451; **Figure 4A**). Field EPSP slopes were significantly affected by stimulation intensity (*F*_10,120_ = 23.032, *P* < 0.0001) and its interaction with treatment (*F*_10,120_ = 2.531, *P* = 0.0084) and LTP (*F*_1,12_ = 12.174, *P* = 0.0045; interaction LTP × stimulation intensity: *F*_10,120_ = 4.970, *P* < 0.0001). Overall, the results indicate that Rho GTPase activation increases excitatory neurotransmission in the hippocampal CA1.

The analysis of fEPSP maximal slopes recorded in the layer III of V1 visual cortex (**Figure 4B**) shows no significant effects of treatment (*F*_1,12_ = 0.027, *P* = 0.8711; interaction treatment × stimulation intensity : *F*_10,120_ = 0.261, *P* = 0.9882). The effects of *LTP* (*F*_1,12_ = 5.291, *P* = 0.0402; interaction LTP × stimulation intensity: *F*_10,120_ = 3.501, *P* = 0.004) were significant, as they were the effects of stimulation intensity (*F*_10,120_ = 19.116, *P* < 0.0001). Apparently, in the layer III of V1 visual cortex, the activation of Rho GTPases does not affect basal glutamatergic neurotransmission and LTP.

All slices reached the criterion for LTP. Responses obtained at baseline intensity were steady both pre and post-TBS. The trend of fEPSP maximal slopes after TBS in hippocampal CA1 and V1 visual cortex are illustrated in **Figures 4C,D**, respectively. Sixty minutes post-TBS, the LTP in hippocampal CA1 area from CNF1-treated mice was significantly higher than that observed in slices from control mice (*P* < 0.05 by ANCOVA, using average fEPSP slope of 10 pre-TBS responses as covariate). On the contrary, the difference was not significant in V1 visual cortex. These results might reflect a plasticizing effect that is selective for hippocampal CA1. However, the findings may also be explained by the different slope of the input/output curves observed in CA1 and therefore do not necessarily reflect a real increase in synaptic plasticity.

The analysis of fEPSP maximal slopes during PPF by ANCOVA for repeated measurements using the slope of the first response as covariate did not show any significant effect of the treatment or its interaction with inter-stimulus interval, neither in hippocampal CA1 (**Figure 4E**) nor in V1 visual cortex (**Figure 4F**).

### THE EFFECTS OF CNF1 DO NOT SHOW HEMISPHERIC DIFFERENCES

**FIGURE 5:**
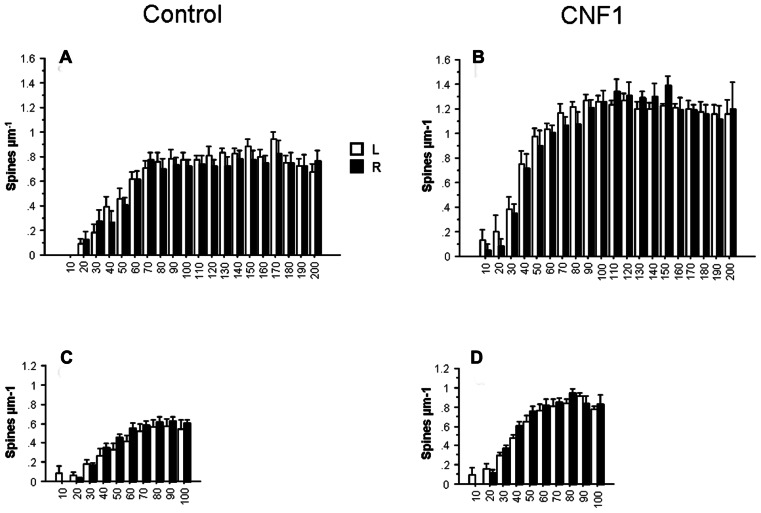
**Effects of Rho GTPase activation on spine density by cerebral hemisphere.** The analysis of spine density by distance from cell soma (μm) in hippocampal CA1 pyramidal neurons of the left (L, *n* = 16) and right (R, *n* = 16) hemisphere. C57BL/6J mice were injected into the right lateral ventricle 10 days before histology. Spine density in basal **(C,D)** and apical **(A,B)** dendrites. Data are mean ± SEM.

The reported effects may be partly explained by a proximity effect, i.e., the tendency of the treatment to selectively affect cell processes that are closer to the injection side. To test this hypothesis, we compared the effects of the treatment in the morphometry of Golgi stained CA1 hippocampal neurons of the two brain hemispheres. If a local effect exists, it should be observed in the injected side. In general, the effect hemisphere was far from being statistically significant in the ANOVA for repeated measurements on dendritic spine densities, dendrite intersections, and dendrite lengths. In **Figures 5A–D** spine density in hippocampal CA1 pyramidal neurons 10 days post-treatment is plotted by treatment and hemisphere in apical (hemisphere: *F*_1,26_ = 0.256, *P* = 0.6171; interaction hemisphere × treatment: *F*_1,26_ = 0.067, *P* = 0.7974) and basal dendrites (hemisphere: *F*_1,27_ = 1.192, *P* = 0.2845; interaction hemisphere × treatment: *F*_1,27_ = 0.049, *P* = 0.8267). The results suggest that the action of CNF1 is widespread and not strictly local or dependent on a gradient of concentration from the injection site.

## DISCUSSION

Here we report that Rho GTPase-dependent plasticity is selectively low in the apical dendrites of layer V pyramidal neurons in primary visual cortex. Although the rates of spine turnover are somewhat different across different cortical regions and pyramidal neurons in primary visual cortex can display a selective stability (Holtmaat et al., 2005; Majewska et al., 2006), spine plasticity is observed in the basal dendrites of these cells, indicating a dendrite selectivity in Rho-dependent plasticity. In addition, the increase in spine density following Rho GTPase activation in the hippocampal CA1 appears to be much higher than that observed in the cortex. Even though the rate of spine turnover cannot be inferred from changes in spine densities, the turnover rate cannot be lower than the rate of increase in spine density. In conclusion, adult brain areas that cannot be explored by *in vivo* multiphoton confocal microscopy display a much higher increase in spine density than the outer neocortex. This finding has several implications.

First, Rho GTPases control the polymerization of the actin cytoskeleton, under the pressure of which dendritic spines are produced and changed (Matsuzaki et al., 2004; Okamoto et al., 2004; Honkura et al., 2008). This mechanism is activity-dependent (Murakoshi et al., 2011). It can be concluded that activity-dependent structural plasticity, which is thought to be associated with learning and memory, can be substantial in selected brain areas.

Second, the selectivity of the increase in the hippocampus and basal dendrites of cortical neurons explains the controversial findings reported using *in vivo* chronic multiphoton imaging when studying experience-dependent changes in dendritic spines. Indeed, according to our findings, this technique explores areas in which this Rho-dependent structural plasticity is relatively poor. The outer neocortex may not be the ideal brain area for exploring the physiological relevance of dendritic spine plasticity in adulthood. Different techniques permitting the *in vivo* study of deeper brain areas will be needed, as well.

Although we cannot rule out the possibility that other mechanisms modify dendritic spines on apical cortical dendrites, regional differences in Rho-GTPase signaling might represent the cause of the stability of cortical circuits. However, since synaptic plasticity follows coincident stimulation at single synaptic buttons, it is also possible that this event is not common in outer cortical layers. As a third explanation, functional synaptic plasticity, which triggers structural plasticity, might be reduced in the apical dendrites of cortical pyramidal neurons. This possibility is suggested by our electrophysiology findings and it was also previously hinted (Frankland et al., 2001).

Whether differences in Rho GTPase-dependent plasticity reflect different distribution of neural activity leading to structural plasticity or functionality of Rho signaling cascade, regional analysis of Rho GTPase-dependent plasticity may provide information about the importance of this process in different areas of the brain. Specifically, our findings might reflect differences in the physiological role of apical dendrites of cortical pyramidal neurons. Functional split of apical and basal dendrites of layer V cortical pyramidal neurons of the barrel cortex was reported (Petreanu et al., 2009). Differences in the degree of structural plasticity (absent in apical, present in basal dendrites) have also been observed in V1 visual cortex pyramidal neurons (Michalon et al., 2012). Although a full characterization of V1 inputs by subcellular areas of the neuron is still to be obtained, it seems that layer II and III and the upper layer IV, which contain apical dendrites of layer V pyramidal neurons, receive long-range connections from the contralateral hemisphere (Rochefort et al., 2009). Thus, the selective structural stability of outer cortical processes might be crucial for complex functions requiring the integration of multimodal inputs.

We also report an increased branching of the hippocampal dendritic tree. The increase was observed after modulation of a physiological pathway in healthy individuals. Thus, such changes might occur in the physiological functioning of the adult brain. This finding suggests that adult activity-dependent neural morphogenesis may not be limited to spines, but it might involve the entire neural tree. The presence of these changes in deep brain structure such as hippocampus, but not in visual cortex, might explain why this phenomenon has never been observed with *in vivo* multiphoton microscopy of adult mice.

Cytotoxic necrotizing factor 1 substantially increases neural connectivity. To our knowledge, no molecule, including synthetic drugs and nervous growth factors, parallels the size of the increase in the number of dendritic spines produced by CNF1 *in vivo*. The increase is equally distributed in the two hemispheres of the brain and was specially observed on the principal dendrite shaft, on oblique dendrites and in the terminal part of the hippocampal neuron. It is worth observing that the effect was seen in adult individuals of a mouse strain displaying excellent performance in hippocampal-dependent learning tasks (Rossi-Arnaud and Ammassari-Teule, 1998). In several disorders, such as those associated with intellectual disability, the impoverishment of the dendritic tree is a consistent stigma (Ramakers, 2002). Reversing this feature by modulation of Rho GTPases might be of therapeutic value (Bongmba et al., 2011). This effect, together with those previously described on learning ability (Diana et al., 2007; De Viti et al., 2010; De Filippis et al., 2012; Borrelli et al., 2013), candidates Rho GTPase modulators as a new avenue for the treatment of several disorders of the central nervous system (Musilli et al., 2013).

## Conflict of Interest Statement

The authors declare that the research was conducted in the absence of any commercial or financial relationships that could be construed as a potential conflict of interest.
